# Characterization of the Molecular Mechanism of the Bone-Anabolic Activity of Carfilzomib in Multiple Myeloma

**DOI:** 10.1371/journal.pone.0074191

**Published:** 2013-09-16

**Authors:** Bo Hu, Yu Chen, Saad Z. Usmani, Shiqiao Ye, Wei Qiang, Xenofon Papanikolaou, Christoph J. Heuck, Shmuel Yaccoby, Bart O. Williams, Frits Van Rhee, Bart Barlogie, Joshua Epstein, Ya-Wei Qiang

**Affiliations:** 1 Myeloma Institute for Research and Therapy, Winthrop P. Rockefeller Cancer Institute, University of Arkansas for Medical Sciences, Little Rock, Arkansas, United States of America; 2 Center for Skeletal Disease Research, Van Andel Research Institute, Grand Rapids, Michigan, United States of America; University of Texas Southwestern Medical Center, United States of America

## Abstract

Carfilzomib, the next generation of proteasome inhibitor, may increase osteoblast-related markers in patients with multiple myeloma, but the molecular mechanism of its effect on mesenchymal stem cell differentiation to osteoblasts remains unknown. Herein, we demonstrated that carfilzomib significantly promoted mesenchymal stem cell differentiation into osteoblasts. In osteoprogenitor cells and primary mesenchymal stem cells from patients with myeloma, carfilzomib induced increases in alkaline phosphatase activity, matrix mineralization, and calcium deposition via Wnt-independent activation of β-catenin/TCF signaling. Using affinity pull-down assays with immunoblotting analysis and immunofluorescence, we found that carfilzomib induced stabilization of both free and active forms of β-catenin in a time- and dose-dependent manner that was not associated with β-catenin transcriptional regulation. Nuclear translocation of β-catenin protein was associated with TCF transcriptional activity that was independent of the effects of GSK3β-activation and of signaling induced by 19 Wnt ligands, 10 Frizzled receptors, and LRP5/6 co-receptors. Blocking activation of β-catenin/TCF signaling by dominant negative TCF1 or TCF4 attenuated carfilzomib-induced matrix mineralization. Thus, carfilzomib induced osteoblast differentiation via Wnt-independent activation of the β-catenin/TCF pathway. These results provide a novel molecular mechanism critical to understanding the anabolic role of carfilzomib on myeloma-induced bone disease.

## Introduction

Multiple myeloma (MM) is characterized by malignant plasma cells infiltrating bone marrow, where they closely interact with mesenchymal stem/stromal cells (MSCs), osteoblasts (OBs), and osteoclasts, triggering osteolytic bone lesions. It has become clear that impaired formation of bone and function of OBs plays a role in development of MM bone lesions. A number of studies have reported fewer OBs and decreased bone formation in MM patients with higher plasma cell infiltration [1]. Myeloma-triggered bone disease is associated with high expression of dickkopf-1 (DKK1) protein in MM cells [2-4] and in the bone marrow microenvironment [5]. DKK1-mediated suppression of Wnt/β-catenin signaling leads to positive regulation of RANKL expression and negative regulation of OB production of osteoprotegerin (OPG), resulting in enhanced osteoclast function [6,7] and in diminished differentiation of OBs from MSCs [8-10]. Preclinical in vivo studies have reported that increasing Wnt/β-catenin signaling by administering anti-DKK1 antibodies, Wnt3a, or LiCl suppresses MM-induced bone loss and MM cell growth [11-15].

β-catenin, a key element in Wnt signaling, plays important roles in regulating differentiation of OBs from MSCs [16] and is involved in myeloma pathogenesis [7,17,18]. β-catenin is located either at the plasma membrane in a complex with cadherins and α-catenin or in the cytoplasm free from cadherin. In response to Wnt ligand binding to Frizzled/LRP5/6 receptor complexes, free β-catenin accumulates in the cytoplasm and then translocates to the nucleus, where it interacts with T-cell factor (TCF)/lymphocyte enhancer factor to modulate activity of target genes [19]. In the absence of Wnt binding, cytoplasmic β-catenin is phosphorylated by casein kinase I and glycogen synthase kinase 3 beta (GSK3β); phosphorylated β-catenin is subsequently ubiquitinated and degraded by the 26S proteasome [20].

Carfilzomib (CFZ) (formerly PR-171) has been investigated as a second-generation proteasome inhibitor and potential anti-MM agent [21]. In clinical studies, MM patients treated with CFZ showed increases in the bone-anabolic marker alkaline phosphatase (ALP) [22], and an in vitro study suggests that CFZ regulates MSC differentiation into OBs [23], but the molecular mechanism underlying CFZ-mediated MSC differentiation is unclear. Thus, we sought to determine the potential roles of CFZ in regulating MSC differentiation and to identify the molecular mechanism(s) involved. Here, we present results demonstrating that CFZ induces MSC differentiation to OBs and identifying CFZ activation of β-catenin/TCF signaling pathways that help regulate MSC differentiation.

## Methods

### Reagents

Clinical-grade CFZ (Onyx Pharmaceuticals, South San Francisco, CA) was dissolved in water (to 10 mM) and stored at -20°C. MG132 cell-permeable proteasome inhibitor was purchased from EMD Chemicals (San Diego, CA). Recombinant Wnt3a (rWnt3a) protein was purchased from R&D Systems (Minneapolis, MN).

### Primary bone-marrow–derived MSCs

The Institutional Review Board Committee of the University of Arkansas for Medical Sciences approved this study. Primary human MSCs used as normal control MSCs (NMSCs) were obtained from the Tulane Center for Preparation and Distribution of Adult Stem Cells (htt://www. som.tulane.edu/gene-therapy/distribute, shtml), and two human MSC samples used as NMSCs were procured from Lonza (www.lonza.com/. Walkersville, MD). MSCs from bone marrow of patients with MM (designated as MMMSCs) were generated using methods previously described [10]; signed Institutional Review Board–approved informed consent forms are kept on record. MSCs were cultured in minimal essential medium with 15% fetal bovine serum. Differentiation assays were performed using cells from passage 2 or 3.

### Cell lines

The following cell lines were used: C3H/10T1/2 (purchased from ATCC, Manassas, VA), immortalized mouse embryonic MSCs; MG63 and Saos-2 (from ATCC), human OB-like cells; and HS5 and HS27A, human bone marrow stromal cells (as described in our previous study [18]). Cells were cultured in Dulbecco’s Modified Eagle Medium (DMEM) (Invitrogen, Carlsbad, CA) containing 10% heat-inactivated fetal bovine serum, penicillin (100 U/ml), streptomycin (100 µg/ml), and 4-mM L-glutamine. Cells were maintained at 37°C and humidified with 95% air and 5% CO_2_.

### Immunoblotting analyses

Following treatment, cell lines and primary MSCs isolated from MM patients were isolated and subjected to immunoblotting analyses as previously described [10]. Proteins were separated by SDS-PAGE and transferred to Immobilon polyvinylidene difluoride membranes (Millipore, Bedford, MA). Anti-β-catenin and anti-non-phosphorylated β-catenin (Millipore) were used as primary antibodies.

### Cell fractionation

Cytosolic and nuclear proteins were prepared by fractionating cell lysates as described [18], with minor modifications. Briefly, cells treated with Wnt-3a or not treated were harvested in hypotonic buffer (10-mM Hepes, pH 7.4; 1-mM MgCl_2_; 0.5-mM CaCl_2_; 1-mM EDTA) and then homogenized by 30 strokes of a Dounce homogenizer. After removing the nuclear pellet, lysates were separated into cytosolic and membrane fractions by ultracentrifugation (100,000 g) for 60 minutes. The nuclear pellets were resuspended in hypotonic buffer containing 0.1% SDS. Proteins from each fraction were subjected to immunoblot analysis.

### GST–E-cadherin binding assays

GST–E-cadherin binding assays were performed as previously described [9]. The β-catenin binding site of E-cadherin was cloned as a GST-fusion protein, and complexes were purified with GST beads. GST–E-cadherin was used to precipitate uncomplexed β-catenin from cell lysates (300 µg); β-catenin was detected by immunoblotting.

### ALP assays

ALP activity was determined as described previously [9]. Briefly, cells were cultured for 96 hours or for 7 days in DMEM with 2% horse serum and CFZ (0–10 nM). Cells were lysed in lysis buffer (150 µl). Absorbance at 402 nm was determined with a Spectra Max340 Microplate Spectrophotometer (Molecular Devices, Sunnyvale, CA). Protein content of lysates was analyzed with the micro-BCA assay kit (Pierce, Rockford, IL).

### Immunofluorescence staining

Immunofluorescence staining was used to identify the active form of β-catenin, as previously described [24]. Briefly, cells were seeded in complete DMEM in 24-well plates in the presence or absence of indicated serial concentrations of CFZ for 14 hours. Cells were then fixed with 3.7% formaldehyde, incubated with anti-β-catenin, and stained with Alexa Fluor 488-labeled goat antibody or Alexa 594-labeled goat antibody (Life Technologies, Grand Island, NY) for 30 minutes at room temperature. Cell nuclei were counterstained with DAPI. Fluorescence was detected with an Axio Observer A1 fluorescence microscope with 10X objective lens (Carl Zeiss Microscopy, Jena, Germany) and images were captured with a SPOT camera (Diagnostic Instruments, Sterling Heights, MI).

### Matrix mineralization

Matrix mineralization was detected with von Kossa staining, using a slight modification of previously described methods [24]. Briefly, cells were cultured in growth medium with indicated concentrations of CFZ. After 21 days of culture, cells were stained with fresh silver nitrate solution (5% w/v) for 30 minutes. An Axio Observer A1 microscope with 40X objective lens and SPOT digital camera was used to capture images. Cells were stained with alizarin red S (ARS) and quantitatively analyzed with methods previously described [24].

### Luciferase reporter assays

To determine TCF activity in response to CFZ treatment, luciferase reporter assays were performed as described previously [9].

### qRT-PCR analysis

Total RNA was isolated (TRIzol reagent; Life Technologies) from cells treated with or without serial concentrations (2 to 20 nM) of CFZ. First-strand cDNA synthesis was performed as previously described [18]. Quantitative PCR (qPCR) was performed with an ABI Prism 7000 sequence-detection system (Life Technologies). PCR primers (Life Technologies) were specific for the following human genes: 19 members of *WNT* family; 10 members of *FZD* family; *DKK1*; *sFRP1*, *-2*, *-3*, and *-4*; *ALPL*; *BMP2*; *CTNNB/β-catenin1*; *OPG*; and *RANKL*; reactions were performed as previously described [10].

### Statistical analyses

Statistical significance of differences between experimental groups was determined with Student’s *t*-test as described previously [10]. Significant *P* values were less than 0.05, based on two-tailed test.

## Results

### CFZ induced increases in free and active forms of β-catenin protein in MSCs

**Figure 1:**
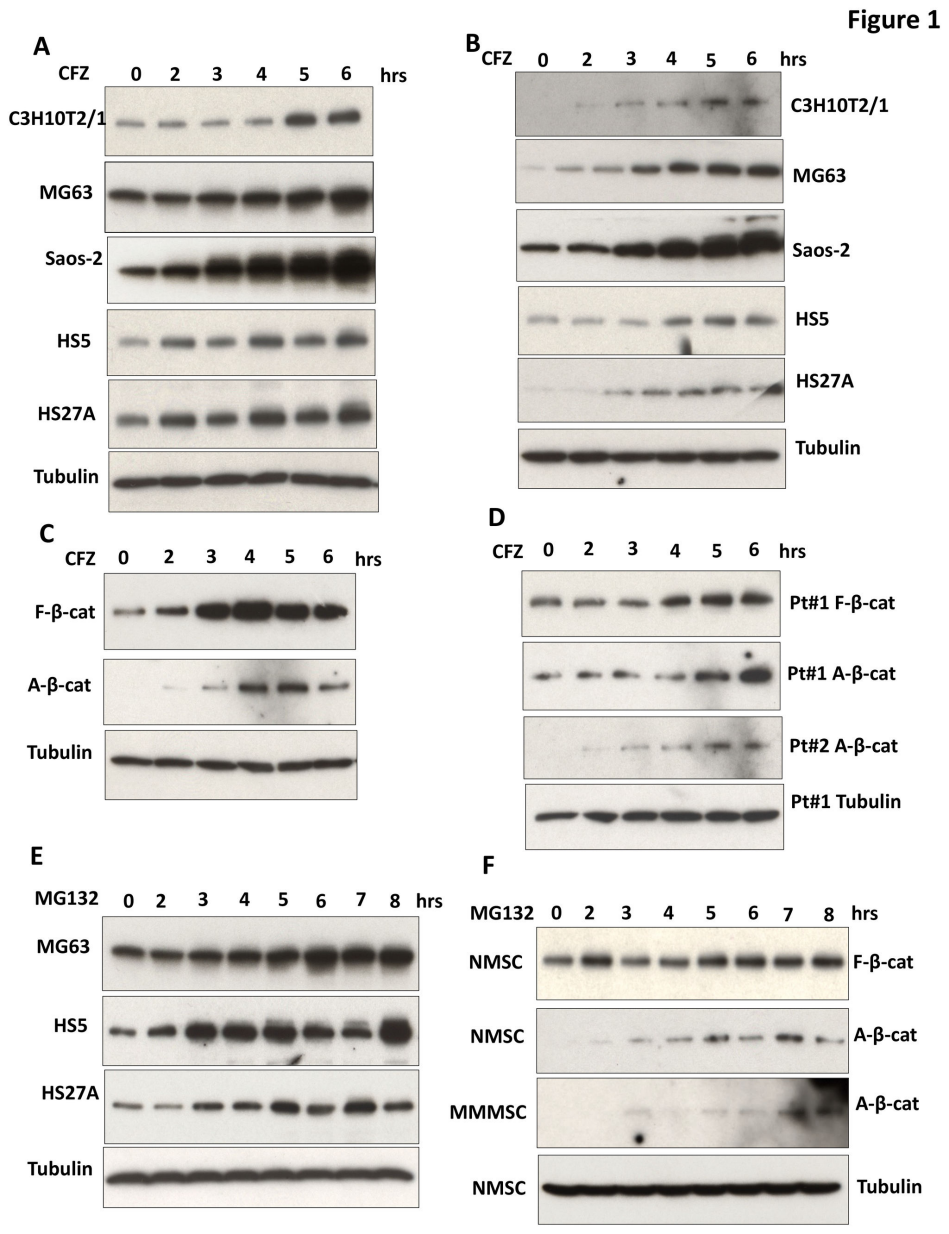
CFZ increased free and active forms of β-catenin in OBs and MSCs. Proteins (500 µg) isolated from cells treated with 20-nM CFZ at serial time points were subjected to GST–E-cadherin pull-down assays and immunoblotting analysis; tubulin was used as a loading control. (**A**) Proteins of indicated cell lines were immunoblotted with anti-β-catenin antibody to detect free β-catenin protein or (**B**) with an antibody that specifically recognizes active, non-phosphorylated β-catenin. The data are representative of three experiments. (**C**) Lysates of human MSCs from a healthy donor or (**D**) from bone marrow of two patients with MM (Pt 1, Pt 2) were treated with 20-nM CFZ for indicated times and were immunoblotted with anti-β-catenin antibody (F-β-cat) or with an antibody that specifically recognizes active, non-phosphorylated β-catenin (A-β-cat). (**E**) Lysates of indicated cell lines treated with 250-nM MG132 were immunoblotted with anti-β-catenin antibody. (**F**) Lysates of cultured human MSCs from a healthy donor (NMSC) or from a patient with MM (MMMSC) were treated with 250-nM MG132 for indicated times and were immunoblotted with anti-β-catenin antibody (F-β-cat) or with an antibody that specifically recognizes active, non-phosphorylated β-catenin (A-β-cat).

**Figure 2:**
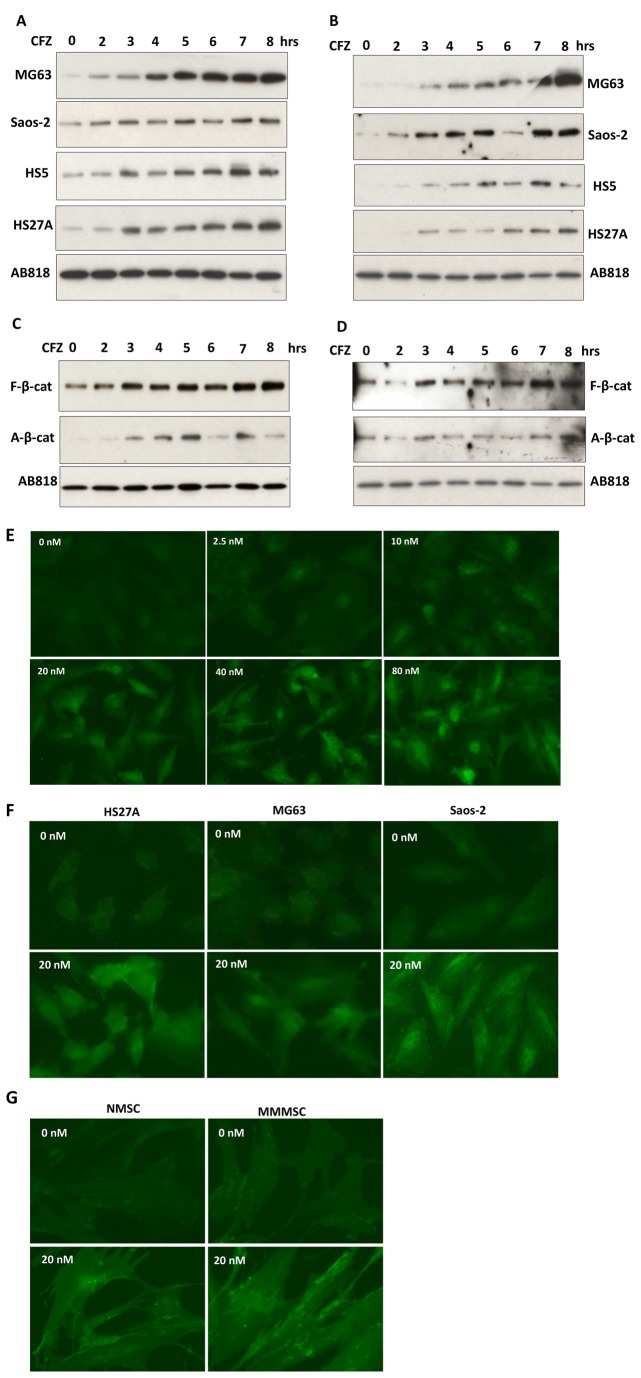
CFZ induced β-catenin accumulation in nuclear fractions of OBs and MSCs. (**A**–**D**) Cells were treated with 20-nM CFZ for indicated times, and nuclear fractions were extracted from cell lysates and subjected to GST-E-cadherin pull-down assays. AB818 protein was measured as a loading control. (**A**) Proteins from indicated OB and MSC cell lines were immunoblotted with anti-β-catenin antibody or (**B**) with an antibody that specifically recognizes active, non-phosphorylated β-catenin. (**C**) Protein from human MSCs from a healthy donor or (**D**) from bone marrow of a patient with MM were immunoblotted with anti-β-catenin antibody (F-β-cat) or with an antibody that specifically recognizes active, non-phosphorylated β-catenin (A-β-cat). (**E**) HS5 cells, (**F**) indicated cell lines, or (**G**) human MSCs from a healthy donor (NMSC) or from bone marrow of a patient with MM (MMMSC) were treated with indicated concentrations of CFZ for 12 hours, and β-catenin in nuclei and cytoplasm of the cells was determined with analysis of immunofluorescence staining, using an antibody specific for active β-catenin. Images were taken with a fluorescence microscope, as described in Methods.

To determine whether CFZ increases β-catenin protein levels in cell lines of OB progenitor cells and MSCs, we first used E-cadherin pull-down assays to separate the pool of free β-catenin from the pool of membrane-bound β-catenin; OBs and MSCs express abundant amounts of E-cadherin, which sequesters β-catenin in the membrane fraction [25] where it is unaffected by Wnt stimulation [9,10,26] or bortezomib treatment [24]. Treating indicated MSC cell lines with CFZ led to an increase in free β-catenin levels in mouse OB progenitor cells (C3H10T1/2), human OB-like cells (MG63 and Saos-2), and human MSCs (HS5 and HS27A) (Figure 1A). Comparable changes were not observed when these cell lysates were immunoblotted for α-catenin and γ-catenin (not shown). Elevated levels of β-catenin protein were observed at 5 hours in C3H10T1/2 cells, and at 2 hours in the other four cell lines (Figure 1A). Maximal β-catenin levels in response to CFZ were observed at 4 hours for HS5 and HS27A cells, 5 hours for C3H10T1/2 cells, and 6 hours for MG63 and Saos-2 cells. Consistent with CFZ-induced preservation of polyubiquitinated forms of β-catenin [27], higher molecular weight forms of β-catenin were seen in CFZ-treated cells. Immunoblotting with antibody specific for the non-phosphorylated form of β-catenin revealed increases in active β-catenin (Figure 1B), with maximal levels observed at 5 hours for C3H10T1/2 cells and at 4 hours for MG63, Saos-2, HS5, and HS27A cells. Immunofluorescence analysis demonstrated increases in β-catenin protein levels in HS5, HS27A, MG63, and Saos-2 cells in response to increased concentrations of CFZ (Figure 2 and Figure S1). Elevated levels of β-catenin protein were observed at 5-nM CFZ, and maximal responses were at 80-nM CFZ. These results indicate that CFZ-mediated proteasome inhibition resulted in increased amounts of active cytosolic β-catenin protein in a time- and dose-dependent manner.

To investigate whether these results obtained in cell lines were representative of those seen in primary samples of MSCs, MSCs isolated from bone marrow of two healthy donors (NMSCs) and from six patients with MM (MMMSCs) were cultured and incubated with CFZ. As with the cell lines, CFZ treatment of NMSCs resulted in increased levels of free β-catenin protein that were evident at 2 hours and peaked at 4 hours (Figure 1C); similar results were observed for the active form of β-catenin. CFZ treatment of MMMSCs from two patients with MM led to increased levels of free β-catenin protein that were evident at 4 hours and in the active form of β-catenin that were evident at 2 hours (Figure 1D). Because the maximal increase in free β-catenin was observed 6 hours after CFZ treatment in MMMSCs from two patients, we treated MSCs from another four patients with MM for 6 hours (cells isolated from these patients were too few to allow treatment with CFZ for multiple time points). CFZ treatment led to obvious increases in free β-catenin in the MSCs from these four additional patients with MM, as compared with the respective untreated MSCs (Figure S4). These data show that CFZ induced increases in levels of free and active forms of β-catenin protein in a time-dependent manner in MSCs from healthy donors and from patients with MM.

To confirm the effects of CFZ on β-catenin in OBs and MSCs, we used a different proteasome inhibitor, MG132, to treat OBs, NMSCs, and MMMSCs. MG132 treatment led to stabilization of free β-catenin in MG63, HS5, and HS27A cell lines in a time-dependent manner (Figure 1E; Saos-2 not shown); similar results were observed for NMSCs (Figure 1F). Levels of the active form of β-catenin increased in response to MG132 in NMSCs and MMMSCs in a time-dependent manner (Figure 1F). Taken together, these results indicate that proteasome inhibition induced stabilization of β-catenin protein in MSCs in a time-dependent manner.

### CFZ induced nuclear accumulation of β-catenin

We next determined whether CFZ treatment led to nuclear accumulation of β-catenin. CFZ induced an increase in levels of free β-catenin (Figure 2A) and in the active form of β-catenin (Figure 2B) in nuclear fractions from MG63, Saos-2, HS5, and HS27A cells; this increase began at 2 hours in Saos-2 and at 3 hours in the other three cell lines, and it persisted until 8 hours. Similar results were seen in NMSCs (Figure 2C) and MMMSCs (Figure 2D). Immunofluorescence analyses of HS5 cells revealed that nuclear accumulation of β-catenin was induced by 12-hour treatment with CFZ at concentrations of 2.5, 10, 20, 40, and 80 nM, with a peak at 80 nM (Figure 2E). At 20-nM CFZ, nuclear β-catenin in HS27A, MG63, and Saos-2 cells was higher than in untreated control cells (Figure 2F); NMSCs and MMMSC showed similar results (Figure 2G). To further confirm the effects of CFZ on stabilization of β-catenin, we extended our studies using escalated concentrations of CFZ for 12 hours. CFZ induced a dose-dependent increase in β-catenin protein in HS27A cells (Figure S1A). Similar results were observed in MG63 (Figure S1B) and in Saos-2 cells (Figure S1C). Finally, CFZ induced increases in β-catenin protein in nuclear fractions of OBs and MSCs, which was confirmed by immunofluorescence analyses using anti-β-catenin antibody with an Alexa 594-labeled goat antibody combined with nuclear DAPI counterstaining. As expected, 12-hour CFZ treatment led to increased β-catenin protein in both cytoplasmic and nuclear fractions in HS5 (Figure S2A), Saos-2 (Figure S2B), and MG63 (Figure S2C) cells and in primary MSCs from two patients with MM (Figure S2D and data not shown). Taken together, these findings indicate that CFZ induced nuclear accumulation of β-catenin in OBs and MSCs in a way that was both time- and dose-dependent.

### CFZ induced TCF transcriptional activity

**Figure 3:**
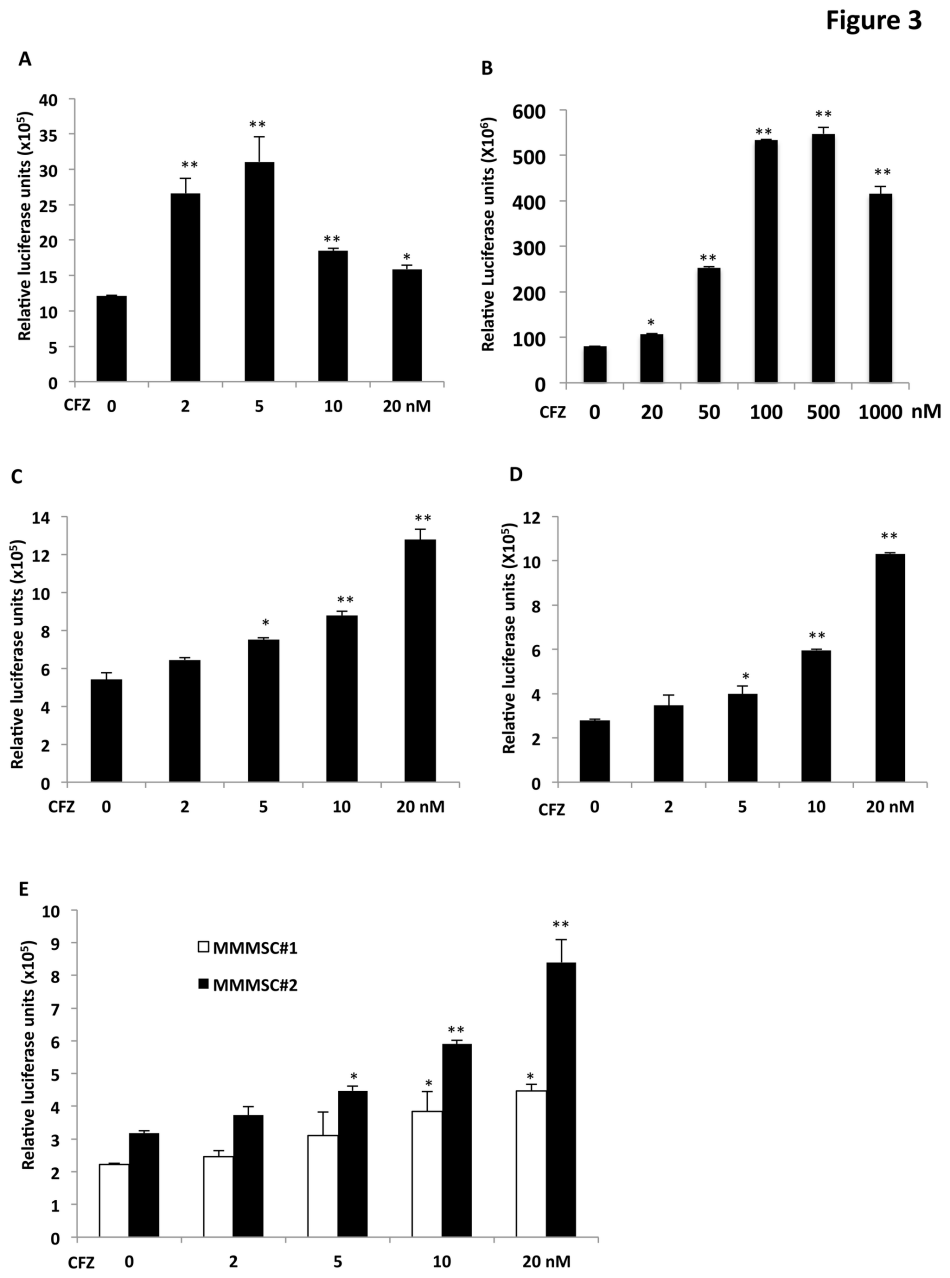
CFZ increased TCF transcriptional activity in OBs and MSCs. (**A**) MG63 cells, (**B**) Saos-2 cells, (**C**) HS5 cells, (**D**) HS27A cells, and (**E**) MSCs from two patients with MM (MMMSC#1, MMMSC#2) were transiently co-transfected with TOPflash (0.5 µg) and pSV-β-galactosidase vector (50 ng; to normalize for transfection efficiency). Cells were treated with indicated concentrations of CFZ for 24 hours, and cell lysates were subjected to luciferase assays. Results represent the mean ± SD (n=3) of triplicate transfection experiments; *P<0.01, **P< 0.001.

Having demonstrated that CFZ significantly induced increases in both free and active β-catenin and induced β-catenin translocation into nuclei of OBs and MSCs, we next investigated whether increased nuclear β-catenin in OBs and MSCs affects TCF transcriptional activity. Using the TOPflash TCF reporter system [9], we found that CFZ treatment significantly induced luciferase activity in MG63, Saos-2, HS5, and HS27A cell lines (Figure 3A–D). Luciferase activity exhibited a dose-dependent response, with maximal responses at 5 nM for MG63 (Figure 3A), 100–500 nM for Saos-2 (Figure 3B), and 20 nM for HS5 (Figure 3C and data not shown) and HS27A (Figure 3D and data not shown) cells; in response to treatment of HS5 and HS27A cells with higher concentrations of CFZ (i.e., 25, 30, 40, and 50 nM), luciferase activities were lower than at 20-nM CFZ, although they were still significantly higher than those of untreated controls (data not shown). Similar results were observed in two samples of MMMSCs, with peak responses at 20-nM CFZ (Figure 3E). MG132 cells also induced luciferase activity in HS27A and MG63 cells (not shown). These results indicate that CFZ stimulated TCF transcriptional activity in a dose-dependent manner in OBs and MSCs.

### CFZ induced differentiation of OB progenitors, NMSCs, and MMMSCs

**Figure 4:**
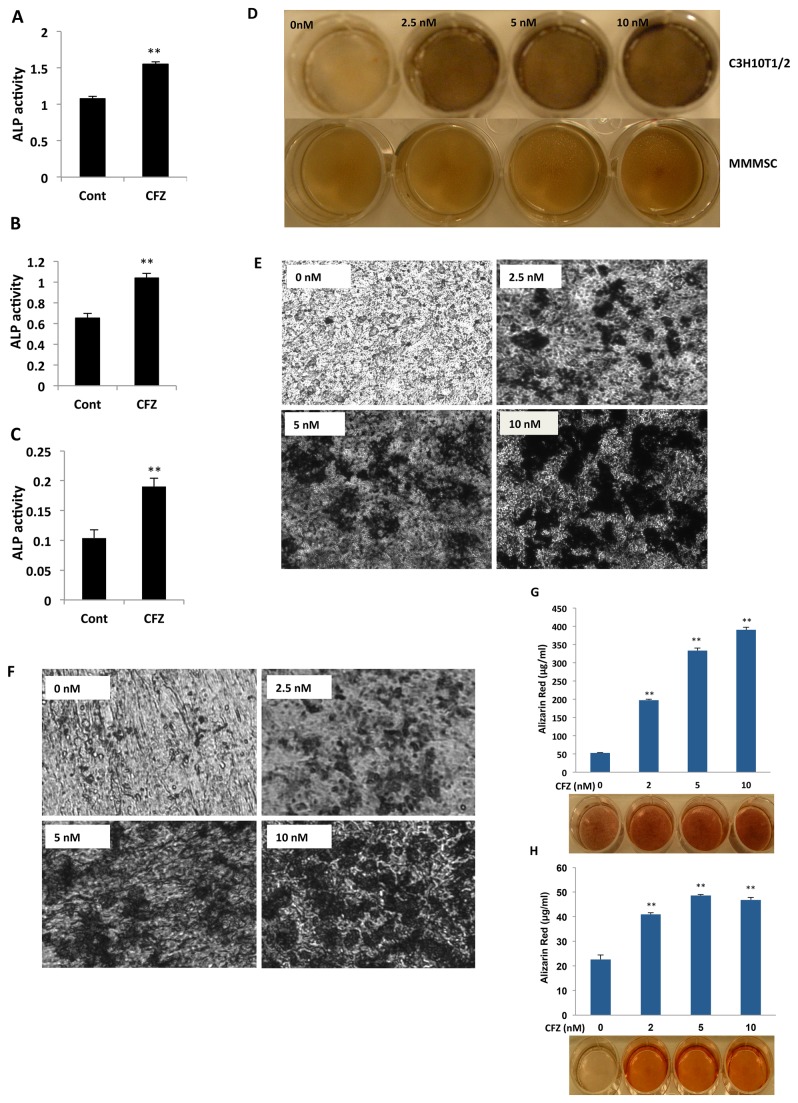
CFZ induced ALP activity and mineralization in MSCs. (**A**) MSCs from a healthy donor or (**B**, **C**) from bone marrow of two patients with MM were cultured in medium in the absence (Cont) or presence of 2-nM CFZ. After 72 hours, cells were lysed and ALP activity determined. Data represent the mean ± SD (n=3) of a representative of three experiments; **P<0.01 versus control. (**D**) C3H10T1/2 cells and MSCs from a patient with MM (MMMSC), (**E**) HS27A cells, and (**F**) MMMSCs were cultured at indicated serial concentrations (0 to 10 nM) of CFZ for 21 days; matrix mineralization was analyzed by von Kossa staining as described in Methods. (**G**) C3H10T1/2 cells and (**H**) MSCs from a patient with MM were cultured with indicated serial concentrations of CFZ for 21 days. Matrix mineralization for calcium deposits was visualized with ARS staining; OD_405_ values were used for quantitative analysis of ARS staining. Results are shown as mean ± SD (n=3); **P<0.01 versus control.

Wnt/β-catenin signaling is required for MSC differentiation to OBs and for bone formation [16], so we initially examined the effects of CFZ on activity of ALP, which is a marker of MSC differentiation to OBs. CFZ treatment of NMSCs resulted in a significant increase in ALP activity (Figure 4A). Similar results were obtained in two samples of MMMSCs (Figure 4B-C). These results indicate that CFZ induced MSC differentiation to OBs in MSCs from healthy donors and from patients with MM.

We next used von Kossa staining to examine effects of CFZ on extracellular matrix mineralization (EMM), which indicates OB function during bone formation. In the presence of CFZ, mineralization was higher in C3H10T1/2 cells and in MMMSCs than in control cells not treated with CFZ (Figure 4D). Micrographs of HS27A (Figure 4E) and MMMSCs (Figure 4F) cell cultures showed that EMM increased as the CFZ concentration increased.

To confirm the effects of CFZ on MSC differentiation, cells were also stained with ARS to detect calcium deposition. We observed significant dose-dependent increases in ARS staining in C3H10T1/2 cells treated with serial concentrations of CFZ, with maximal effects observed at 10-nM CFZ (Figure 4G); similar results were observed in two samples of MMMSCs (Figure 4H). Thus, CFZ treatment of MSCs and MMMSCs from patients with MM is capable of stimulating in vitro EMM, indicating differentiation to functional OBs.

### CFZ-activated β-catenin/TCF signaling occurs independently of upstream targets of β-catenin

**Figure 5:**
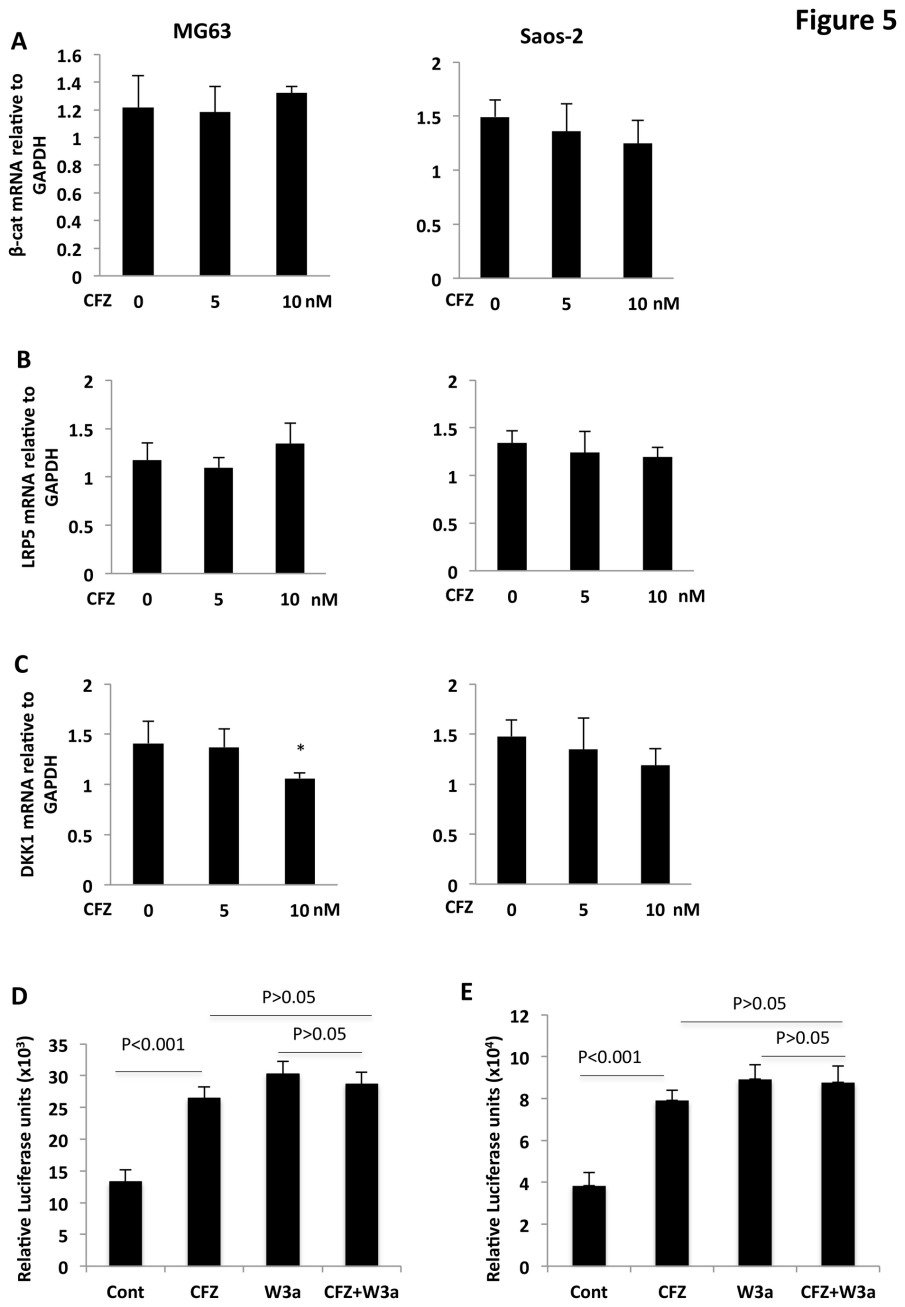
Effects of CFZ on components of Wnt/β-catenin signaling. (**A**, **B**, **C**) MG63 (left) and Saos-2 (right) cells were cultured in medium with indicated concentrations of CFZ, and total RNA was isolated and assayed for synthesis of cDNA. qRT-PCR analysis was performed to measure mRNA expression of indicated genes. Results are shown as mean ± SD (n=3); *P<0.01, versus control. (**D**) MG63 cells and (**E**) MSCs from MM patients transfected with TOPflash plasmid DNA were treated for 24 hours with 20-nM CFZ in the absence or presence of 100-ng/ml Wnt3a or were treated with Wnt3a alone. Lysates were harvested and subjected to luciferase assays. Results are shown as mean ± SD (n=3).

To investigate whether CFZ-stimulated activation of β-catenin/TCF was involved in regulating transcription of β-catenin, we analyzed the effects of CFZ on β-catenin mRNA levels. CFZ treatment did not result in changes in β-catenin gene expression in MG63 and Saos-2 cell lines (Figure 5A) or in MMMSCs (not shown). These results, together with the CFZ-induced increase in β-catenin protein (Figures 1–2), confirm that CFZ regulates β-catenin protein at the posttranslational level, independent of transcription.

We next examined whether CFZ directly regulates extracellular components of the Wnt signaling pathway; proteasome inhibitors increase BMP-2 expression in mouse OBs [28], and BMP-2 induces canonical Wnt signaling through autocrine activation of Wnt3a in C3H10T1/2 cells [29]. Treating Saos-2 and MG63 cells with 5- and 10-nM CFZ did not significantly induce expression of *LRP5/6* (Figure 5B), but it did result in downregulation of *DKK1* in one of two MSC cell lines (Figure 5C). Because addition of exogenous DKK1 inhibits Wnt/β-catenin signaling in MSCs [9], we next examined whether the CFZ-induced decrease in DKK1 mRNA had an effect on Wnt signaling. Treating MG63 cells with recombinant Wnt3a protein and CFZ did not result in higher levels of free β-catenin (not shown) or of TCF transcriptional activity (Figure 5D), compared to the effects of CFZ. Similarly, CFZ and Wnt3a had no significant synergistic effect on TCF activity in MSCs isolated from MM patients. Thus, CFZ-activated β-catenin/TCF signaling occurred independent of regulating extracellular Wnt ligands and co-receptors.

Given that CFZ treatment resulted in decreased Dkk1 mRNA levels, we asked whether DKK1 could block the effects of CFZ on MSC differentiation. To address this question, we assessed the effects of DKK1 on CFZ-induced ALP activity in the absence or presence of Wnt3a (as positive control for indicating function of DKK1) in the differentiation medium for primary MSCs from two patients with MM. Administration of rDKK1 protein (200 ng/ml) did not significantly induce a decrease in ALP activity in the MSCs in the culture medium in the presence of CFZ (Figure S3A), compared with CFZ alone, but it significantly suppressed endogenous Wnt-induced ALP activity (DKK1 alone). These results indicate that DKK1 does not block CFZ-induced MSC differentiation into OBs. Additionally, MSCs cultured in medium containing CFZ, DKK1, and Wnt3a had ALP activity levels similar to those cultured in medium containing Wnt3a alone but significantly higher than in those cultured in medium with Wnt3a and DKK1 (without CFZ). Similar results were seen in MSCs from a second patient (Figure S3B). Taken together, these results indicate that CFZ might overcome the negative effects of DKK1 on differentiation of MSCs and that DKK1 does not block the effects of CFZ on MSC differentiation.

### Blocking TCF function abrogated CFZ-induced MSC differentiation

**Figure 6:**
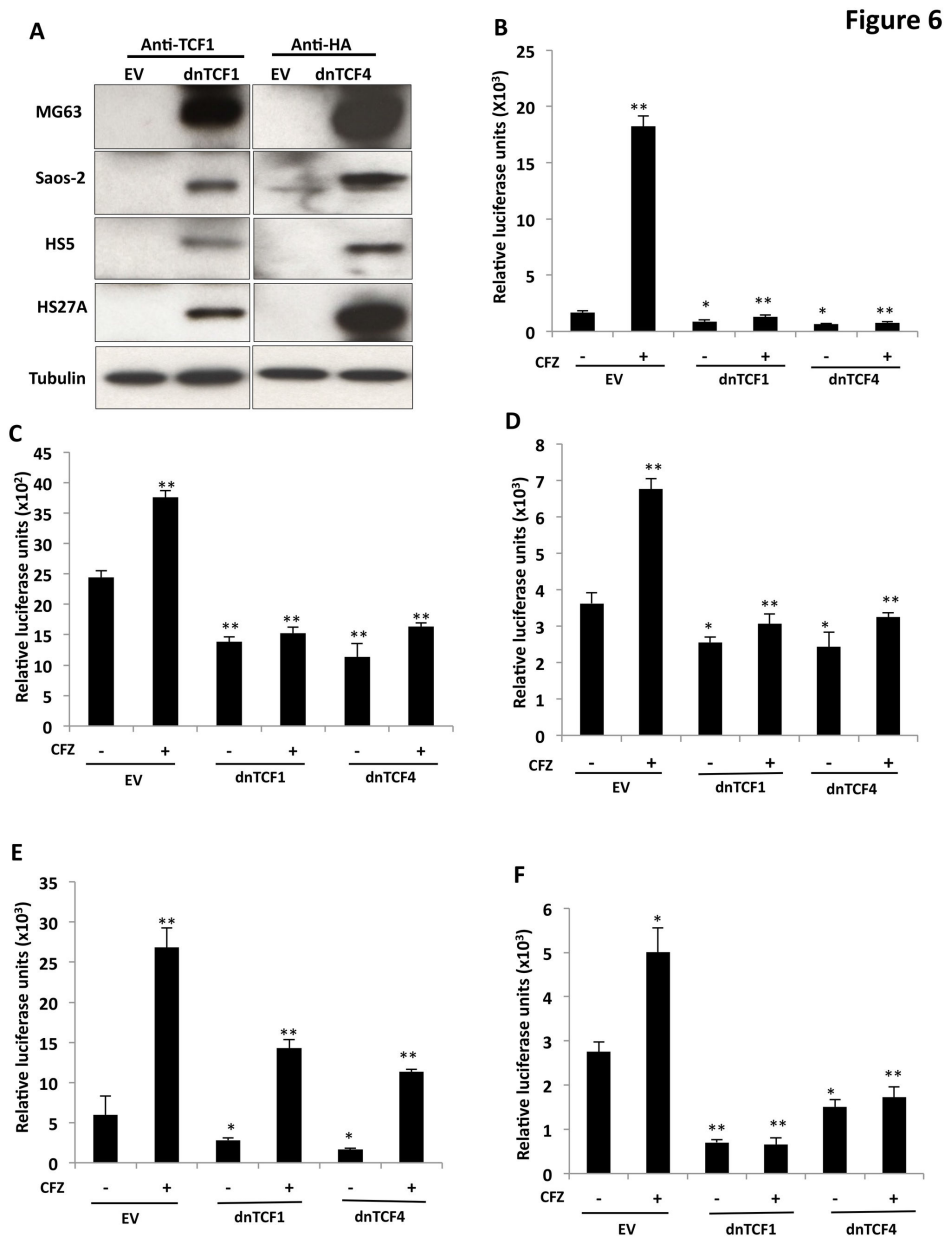
Expression of dnTCF1 or dnTCF4 attenuated CFZ-induced TCF transcriptional activity in MSCs. (**A**) Indicated cells transfected with empty vector (EV), dnTCF1, or dnTCF4 plasmid DNA were lysed, and proteins were subjected to immunoblotting analysis with anti-TCF1 or anti-HA antibodies to measure dnTCF1 and dnTCF4 proteins. (**B**) MG63 cells, (**C**) Saos-2 cells, (**D**) HS5 cells, (**E**) HS27A cells, and (**F**) MSCs from bone marrow of a patient with MM were co-transfected with TOPflash plasmid DNA and either empty vector (EV), dnTCF1, or dnTCF4 plasmid DNA. Cells were incubated in the absence or presence of 5-nM CFZ. Lysates were analyzed for luciferase activity. Data are presented as mean ± SD (n=3); *P<0.01, **P< 0.001 versus controls.

Given that CFZ activated β-catenin/TCF activity and induced MSC differentiation, we next investigated whether TCF signaling was required and/or sufficient for the action of CFZ. Because our previous studies demonstrated that both *TCF1* and *TCF4* were most abundant in OBs and MSCs [24], we assessed the effects of blocking TCF activity in MG63, Saos-2, HS5, HS27A, and MMMSCs by transfecting cells with constructs that express dominant-negative forms of TCF1 (dnTCF1) or TCF4 (dnTCF4) or with empty vector (control). dnTCF4 constructs were HA-tagged and were readily detected by anti-HA antibody in transfected cells; anti-TCF1 antibody was used to detect dnTCF1 (Figure 6A). CFZ significantly induced TCF transcriptional activity in MG63 cells carrying the empty vector, but TCF transcriptional activity was significantly inhibited in cells expressing dnTCF1 or dnTCF4 (Figure 6B). It should be noted that TCF activity in untreated MG63 cells expressing dnTCF1 or dnTCF4 was significantly lower than in cells carrying the empty vector, suggesting that expression of dnTCF1 and dnTCF4 attenuated endogenous TCF activity due to expression of several Wnt ligands in the cells. In similar experiments, expression of dnTCF1 or dnTCF4 significantly diminished CFZ-induced TCF transcriptional activity in Saos-2 (Figure 6C), HS5 (Figure 6D), and HS27A (Figure 6E) cell lines. Importantly, expression of dnTCF1 or dnTCF4 significantly blocked both endogenous and CFZ-induced TCF activity in MMMSCs (Figure 6F). These results indicate that expression of dnTCF1 or dnTCF4 functionally inhibited endogenous and CFZ-induced TCF activity.

**Figure 7:**
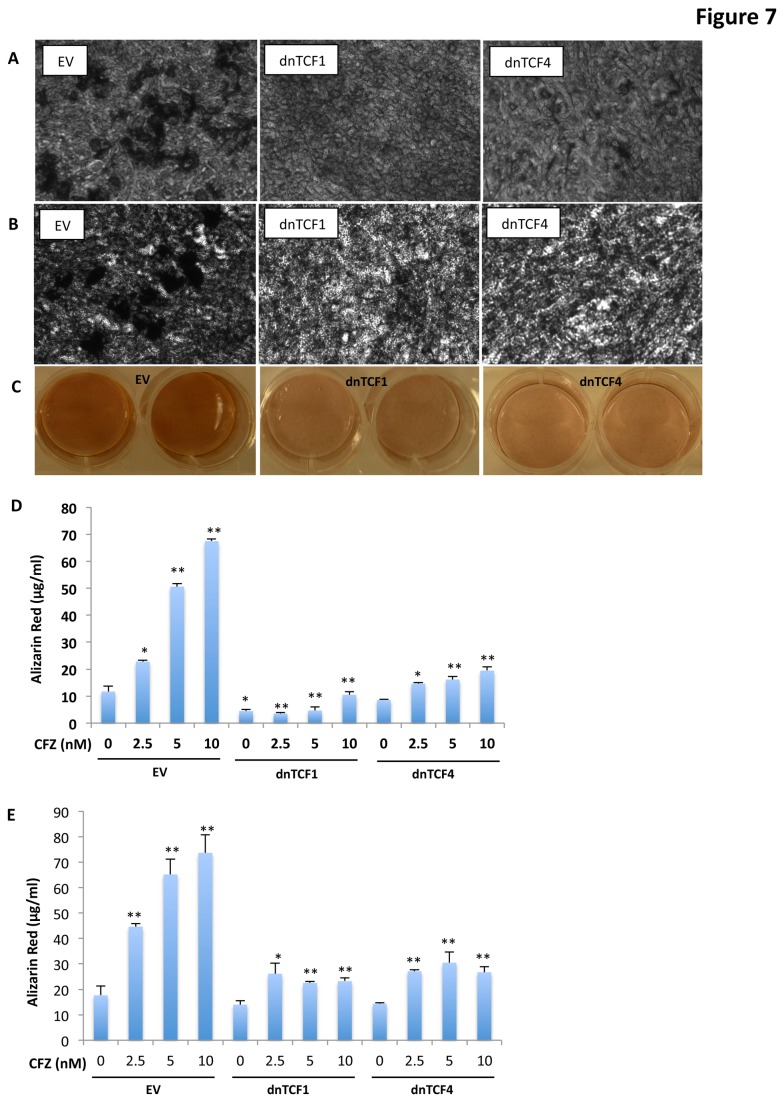
Blocking TCF transcriptional activity decreased CFZ-induced MSC differentiation. (**A**) HS27A and (**B**) C3H10T1/2 cells were transfected with empty vector (EV), dnTCF1, or dnTCF4 plasmid DNA for 48 hours. Cells were cultured in growth medium in the presence of 2.5-nM CFZ for 21 days. Calcium deposition in the cellular matrix was analyzed by (**A**, **B**) von Kossa staining or (**C**) ARS staining in 12-well plates (duplicate wells are shown). (**D**) HS27A cells and (**E**) MSCs from a patient with MM were transfected with indicated plasmids and cultured in growth medium in the presence of serial concentrations of CFZ for 21days. Calcium deposition in the cellular matrix was analyzed by ARS staining; OD_405_ values were used for quantitative analysis of ARS staining. Results are shown as mean ± SD (n=3); *P<0.001, ** *P*<0.0001 versus control.

We next sought to determine whether CFZ-induced MSC differentiation was attenuated when TCF activity was blocked. In response to CFZ treatment, HS27A (Figure 7A) and C3H10T1/2 (Figure 7B) cells transfected with dnTCF1 or dnTCF4 constructs showed attenuated matrix mineralization (based on von Kossa analyses), which was evidenced by decreased cluster sizes and numbers of calcium deposits relative to those of control cells carrying empty vector. Similar results were seen with ARS staining in C3H10T1/2 cells (Figure 7C). Quantitative analysis of ARS staining showed that CFZ treatment significantly induced dose-dependent increases in ARS staining in HS27A cells, and this effect was significantly diminished in cells expressing dnTCF1 and dnTCF4 (Figure 7D). Quantitative ARS staining of MMMSCs treated with CFZ showed that dnTCF1 and dnTCF4 significantly blocked CFZ-induced mineralization (Figure 7E). These results suggest that CFZ-induced OB differentiation from OB progenitors and MSCs occurs in part through activation of β-catenin/TCF signaling.

## Discussion

Suppression of the ability of MSCs to differentiate into OBs that occurs as a result of their interactions with myeloma cells is a fundamental part of the cellular basis for MM-triggered decreases in bone formation. Several in vitro and in vivo studies have shown that bortezomib, a first-generation proteasome inhibitor, is associated with inducing MSCs to differentiate into OBs [24,30,31] and with increases in markers of bone formation in MM patients [32,33]. CFZ, an irreversible next-generation proteasome inhibitor, has been reported to be effective for patients with relapsed and/or refractory MM [34,35]. Preclinical studies provided evidence that CFZ was active against bortezomib-resistant myeloma cells [36], and clinical studies have shown increases in bone-anabolism marker ALP in MM patients treated with CFZ [22]. Recently, a preclinical study demonstrated that CFZ induces MSC differentiation into OBs and increased bone volume in the 5TMG1 myeloma mouse model [23]. However, the molecular mechanism by which CFZ contributes to inducing MSCs to differentiate into bone-forming OBs has not yet been determined. The present studies confirm the role of CFZ in MSC differentiation and describe a molecular mechanism by which CFZ promotes MSC differentiation to OBs and likely alleviates MM-triggered bone disease.

Initial studies demonstrated that CFZ enhanced activation of a β-catenin functional signaling pathway, as evidenced by accumulation of the transcriptionally active form of β-catenin and the uncomplexed, ubiquitinated form of β-catenin in the cytosol in mouse OB progenitor cells (C3H10T1/2 cell line), human OB-like cells (Saos-2 and MG63 cell line), NMSCs, and MMMSCs from six patients (Figure 1); similar results were observed when cells were treated with another proteasome inhibitor (i.e., MG132). Similar to the effects of bortezomib on β-catenin protein in OBs and MSCs [24], CFZ induced stabilization of free and active forms of β-catenin. Accumulation of β-catenin in the cytoplasm led to its nuclear translocation. Moreover, CFZ treatment resulted in functional activation of transcription factor TCFs. Finally, CFZ-stimulated TCF transcriptional activity could be suppressed by expression of a dominant-negative TCF construct. These results suggest that CFZ, similar to bortezomib, promotes the β-catenin/TCF signaling pathway in MSCs.

It was previously reported that the β-catenin/TCF pathway is required for promoting MSC differentiation into OBs [26,29], and it was of interest to determine whether activation of this pathway was involved in CFZ-induced MSC differentiation. In the present studies, it was observed that CFZ could induce MSCs to differentiate to OBs, as evidenced by increases in ALP activity and matrix mineralization in MSC cultures. These results are similar to the effects of Wnt3a [9,14], which stimulates ALP activity in a variety of MSCs [26,29]. These results are consistent with previous reports in which bortezomib induced MSC differentiation [37] and also with correlative studies of primary MM [38]. CFZ-induced MSC differentiation via activation of β-catenin/TCF signaling is further supported by evidence that CFZ-induced OB differentiation was attenuated by blocking this pathway with expression of dnTCF1 and dnTCF4 to diminish TCF activity. These results suggest that an important role in regulating MSC differentiation can be attributed to increased activation of β-catenin/TCF transcriptional activity that results from inhibition of proteasome-mediated degradation of β-catenin by CFZ.

Although CFZ clearly stimulates activation of β-catenin/TCF signaling, it does so without regulating upstream targets of β-catenin, which are required for Wnt3a-initiated activation of the pathway. This conclusion is supported by several observations. First, CFZ did not influence expression of extracellular signaling components, including multiple *Wnt ligands*, *LRP5/6*, and the 10 members of the *Frizzled*/*FZD* receptors (data not shown). Second, CFZ did not synergize with Wnt3a to stimulate TCF activity. Third, blocking GSK3β activity (with lithium chloride, a GSK3β inhibitor) did not elicit a greater level of TCF transcriptional activity in the presence of CFZ in MG63 and Saos-2 cells (data not shown). Although CFZ decreased *DKK1* mRNA levels, CFZ did not synergize with Wnt3a-induced TCF activity, indicating that its effects on stabilization of β-catenin are sufficient to overcome DKK1 inhibition of the canonical Wnt signaling in MSCs and OBs. Finally, CFZ did not affect β-catenin gene expression, implying that CFZ induces activation of β-catenin/TCF activity by blocking β-catenin degradation. CFZ activates Wnt-independent β-catenin/TCF signaling with a mechanism similar to that used by bortezomib [24].

The data presented here suggest that CFZ may be able to bypass DKK1’s suppressive effects on Wnt signaling by directly inhibiting the proteasome and leading to Wnt-independent β-catenin/TCF signaling in bone cells. We previously reported that thalidomide, lenalidomide, and dexamethasone—but not bortezomib—induce *DKK1* expression in MM cells [39,40]. It is now recognized that glucocorticoids also can activate *DKK1* expression in OBs and may contribute to osteoporosis induced by chronic exposure to this class of drug [41]. Interestingly, CFZ treatment of MMMSCs leads to *DKK1* suppression. This observation is in agreement with previous studies revealing that bortezomib treatment results in decreased levels of DKK1 in serum of MM patients [42-44]. It should be noted that CFZ induced a significant decrease in *DKK1* mRNA in MG63 cells but did not show statistically significant decrease in Saos-2 cells. CFZ suppressed growth of MG63 cells but not of Saos-2 cells, which do not express p53, which may account for the drug’s different effects on *DKK1* expression in the two cell lines. It has been reported that *DKK1* expression is dynamically regulated by altering cell cycle progression in human MSCs [45]. Further studies are needed to elucidate the mechanisms underlying the different effects of CFZ on *DKK1* expression in these two cell lines. Because glucocorticoids, thalidomide, and lenalidomide are used to treat MM, combining CFZ with these compounds may be a potential benefit due to its ability to overcome the potential negative side-effects on bone that result from drug-induced activation of *DKK1.*

In conclusion, these studies have revealed that CFZ-induced activation of β-catenin/TCF signaling contributes to promoting OB progenitors and MSCs to differentiate to OBs. CFZ activates β-catenin/TCF signaling via inhibition of proteasome-mediated degradation of β-catenin, which occurs independent of Wnt ligand, Frizzled/FZD receptors, and LRP5/6 co-receptor. These data provide mechanistic insights into CFZ’s bone-anabolic effects and a rationale for its use in treating Wnt/β-catenin–mediated suppression of MSC differentiation.

## Supporting Information

Figure S1 **CFZ induced increases in active β-catenin protein in nuclei and cytoplasm of MSCs.** HS27A (**A**), MG63 (**B**) and Saos-2 (**C**) cells were treated with indicated concentrations of CFZ for 12 hours. The active form of β-catenin in cells nuclei and cytoplasm was examined by immunofluorescence staining, using an antibody specific for active β-catenin and an FITC-labeled goat anti-mouse secondary antibody. Images were taken with an Axio Observer A1 fluorescence microscope with 10X objective lens (Carl Zeiss Microscopy, Jena, Germany) and SPOT camera (Diagnostic Instruments, Sterling Heights, MI).
(TIF)

Figure S2 **CFZ induced increases in β-catenin protein in nuclei and cytoplasm of MSCs.** HS5 (**A**), Saos-2 (**B**), MG63 (**C**), and human primary MSCs from bone marrow of one MM patient (**D**) were treated with indicated concentrations of CFZ for 12 hours and fixed as described in Methods. β-catenin protein in nuclei and cytoplasm of the cells was examined by immunofluorescence staining, using an antibody specific for β-catenin protein and a red-fluorescent-dye-labeled goat anti-mouse antibody. Nuclei were counterstained with DAPI. Images were taken and analyzed as described in Figure S1.
(TIF)

Figure S3 **DKK1 did not block CFZ-induced increase in ALP activity in MSCs.** Human primary MSCs isolated from bone marrow of two patients with MM (**A**, **B**) were cultured in medium alone (Cont.) or with 2-nM CFZ; DKK1; CFZ and DKK1; Wnt3a; Wnt3a and DKK1 (positive control indicating DKK1 function); or CFZ, DKK1, and Wnt3a. After 72 hours, cells were lysed and ALP activity determined as described in Methods. Data represent the mean ± SD (n=3) of three experiments. Statistical analyses to determine significance for each treatment group compared with control or compared among the treated groups was performed as described in Methods.
(TIF)

Figure S4 **CFZ increased the free form of β-catenin in MSCs from patients with MM.** MSCs from four patients with MM were treated with 20-nM CFZ for 6 hours, and proteins (500 µg) were isolated and subjected to GST-E-cadherin pull-down assays and immunoblotting analysis with anti-β-catenin antibody as described in Methods; tubulin was used as a loading control.
(TIF)
