# Supplementary figures and images for: Characterization of the Molecular Mechanism of the Bone-Anabolic Activity of Carfilzomib in Multiple Myeloma
Source: PLoS One. 2013 Sep 16;8(9):e74191. doi: 10.1371/journal.pone.0074191 (PMC3774816; doi:10.1371/journal.pone.0074191)

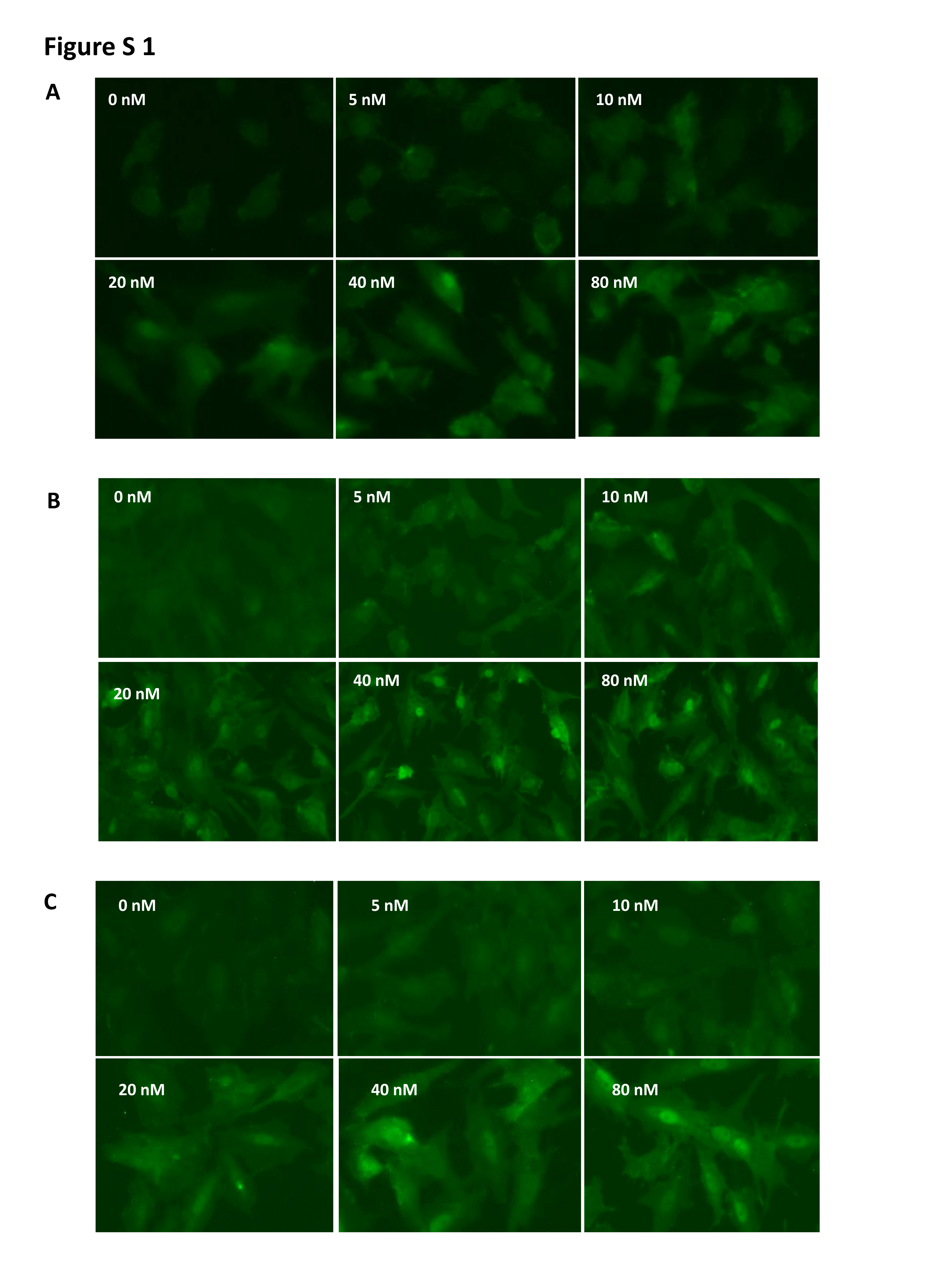

Supplement: Figure S1 — CFZ induced increases in active β-catenin protein in nuclei and cytoplasm of MSCs. HS27A (A), MG63 (B) and Saos-2 (C) cells were treated with indicated concentrations of CFZ for 12 hours. The active form of β-catenin in cells nuclei and cytoplasm was examined by immunofluorescence staining, using an antibody specific for active β-catenin and an FITC-labeled goat anti-mouse secondary antibody. Images were taken with an Axio Observer A1 fluorescence microscope with 10X objective lens (Carl Zeiss Microscopy, Jena, Germany) and SPOT camera (Diagnostic Instruments, Sterling Heights, MI). (TIF) [file pone.0074191.s001.tif]

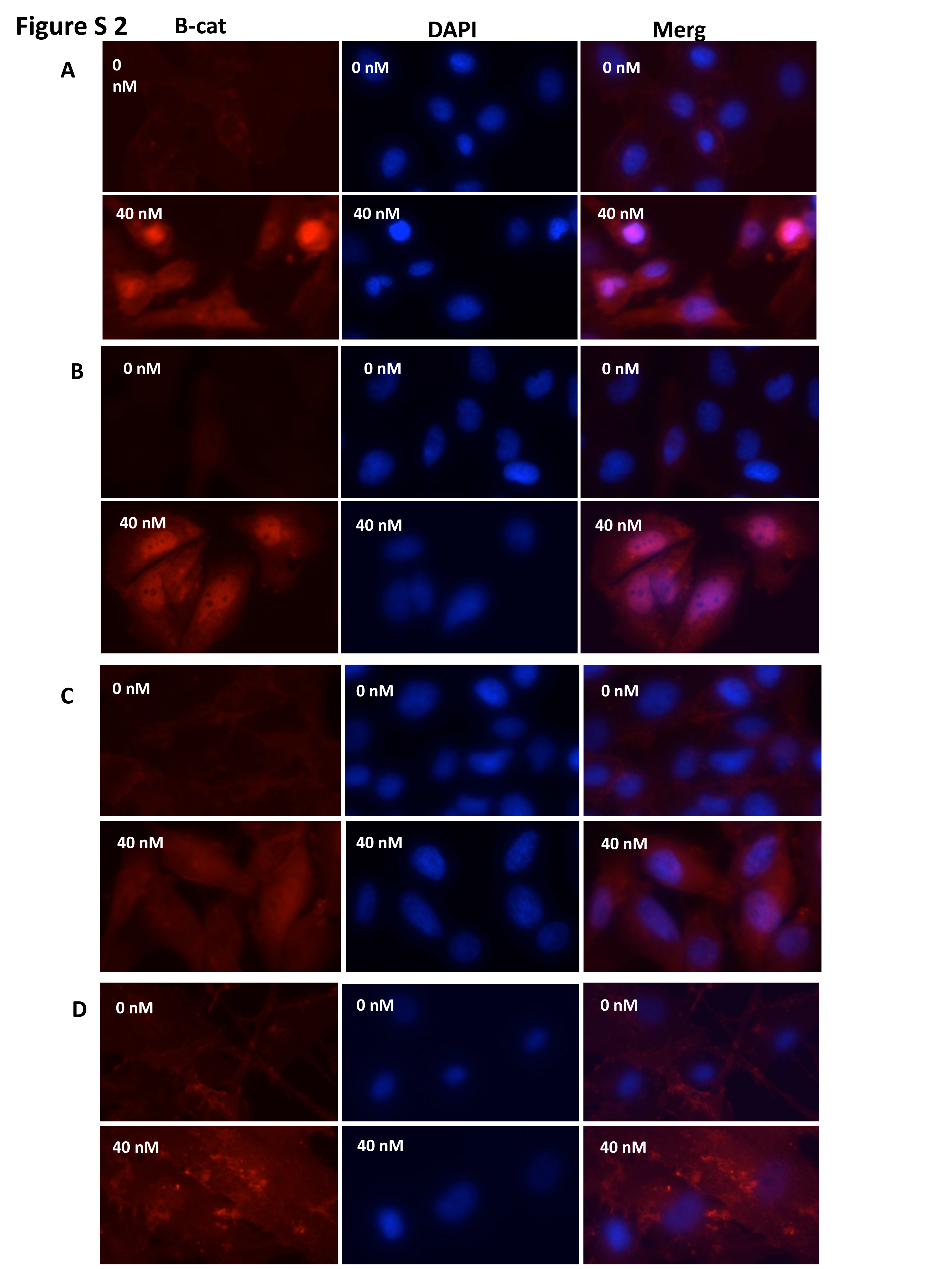

Supplement: Figure S2 — CFZ induced increases in β-catenin protein in nuclei and cytoplasm of MSCs. HS5 (A), Saos-2 (B), MG63 (C), and human primary MSCs from bone marrow of one MM patient (D) were treated with indicated concentrations of CFZ for 12 hours and fixed as described in Methods. β-catenin protein in nuclei and cytoplasm of the cells was examined by immunofluorescence staining, using an antibody specific for β-catenin protein and a red-fluorescent-dye-labeled goat anti-mouse antibody. Nuclei were counterstained with DAPI. Images were taken and analyzed as described in Figure S1. (TIF) [file pone.0074191.s002.tif]

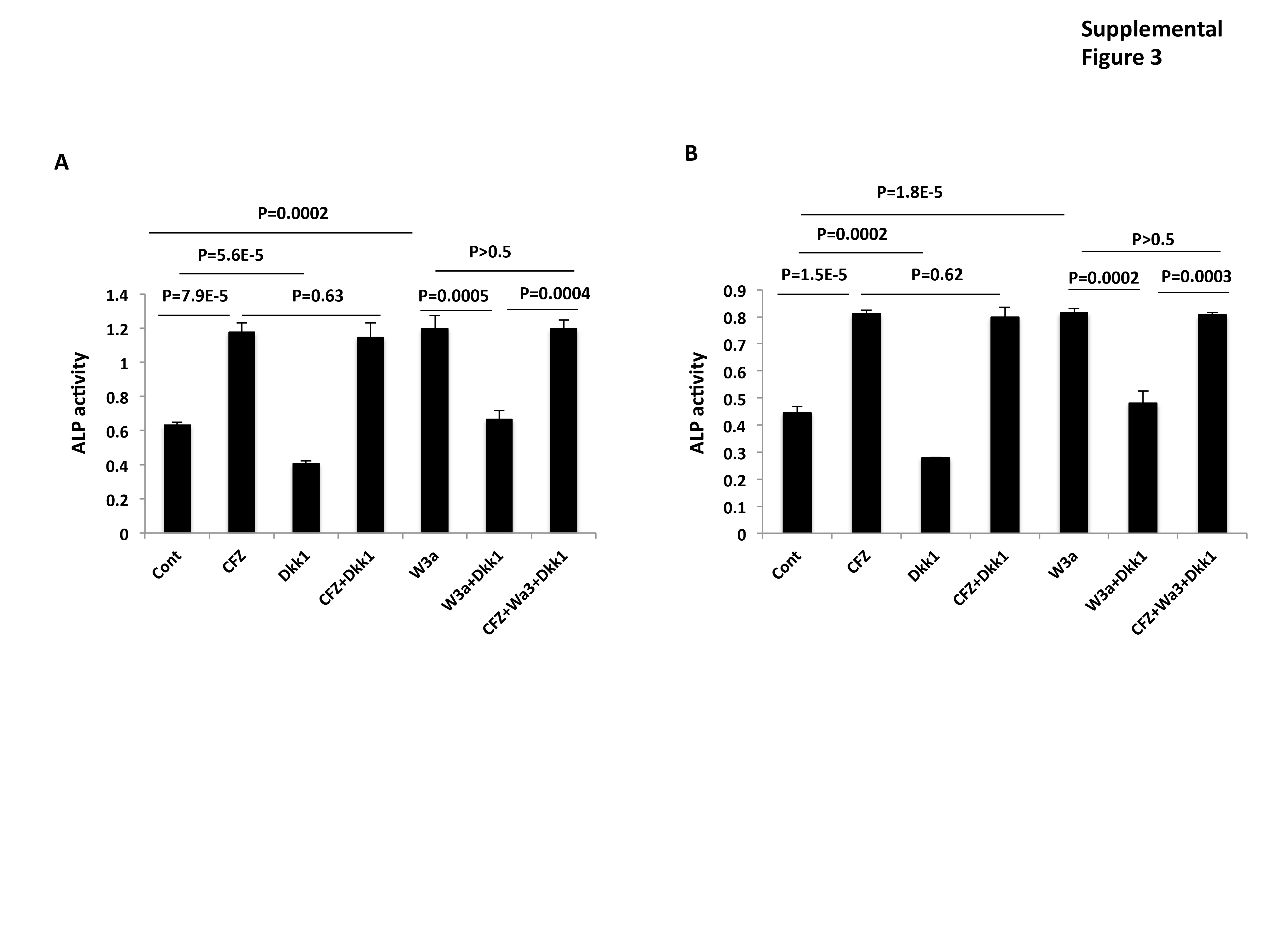

Supplement: Figure S3 — DKK1 did not block CFZ-induced increase in ALP activity in MSCs. Human primary MSCs isolated from bone marrow of two patients with MM (A, B) were cultured in medium alone (Cont.) or with 2-nM CFZ; DKK1; CFZ and DKK1; Wnt3a; Wnt3a and DKK1 (positive control indicating DKK1 function); or CFZ, DKK1, and Wnt3a. After 72 hours, cells were lysed and ALP activity determined as described in Methods. Data represent the mean ± SD (n=3) of three experiments. Statistical analyses to determine significance for each treatment group compared with control or compared among the treated groups was performed as described in Methods. (TIF) [file pone.0074191.s003.tif]

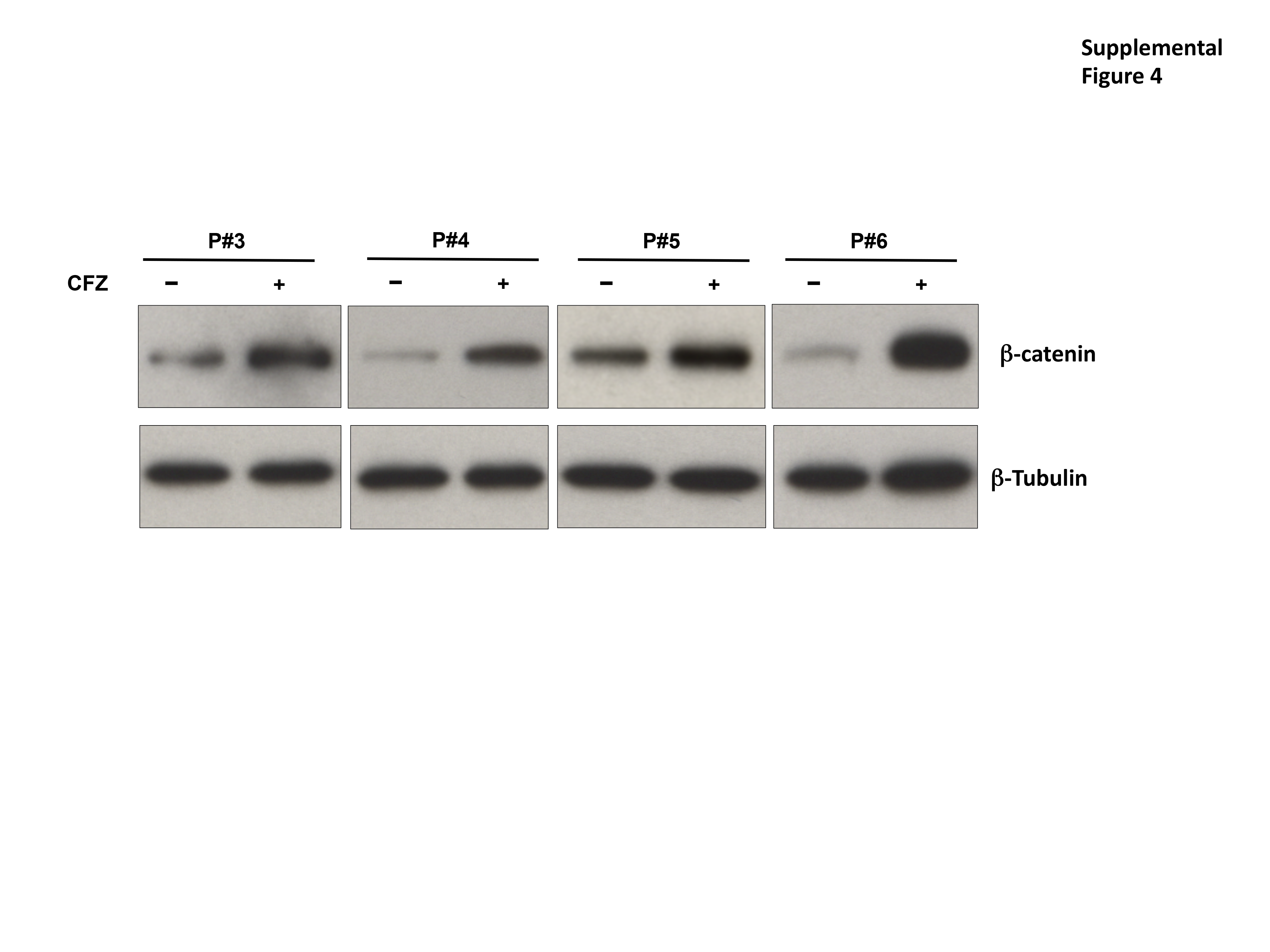

Supplement: Figure S4 — CFZ increased the free form of β-catenin in MSCs from patients with MM. MSCs from four patients with MM were treated with 20-nM CFZ for 6 hours, and proteins (500 µg) were isolated and subjected to GST-E-cadherin pull-down assays and immunoblotting analysis with anti-β-catenin antibody as described in Methods; tubulin was used as a loading control. (TIF) [file pone.0074191.s004.tif]
